# Leptin stimulates autophagy/lysosome-related degradation of long-lived proteins in adipocytes

**DOI:** 10.1080/21623945.2019.1569447

**Published:** 2019-02-08

**Authors:** Nir Goldstein, Yulia Haim, Pamela Mattar, Sapir Hadadi-Bechor, Nitzan Maixner, Peter Kovacs, Matthias Blüher, Assaf Rudich

**Affiliations:** a Department of Clinical Biochemistry and Pharmacology, Ben-Gurion University of the Negev, Beer-Sheva, Israel; b The National Institute of Biotechnology in the Negev, Ben-Gurion University of the Negev, Beer-Sheva, Israel; c Department of Medicine, University of Leipzig, Leipzig, Germany

**Keywords:** Adipocytes, leptin, macro-autophagy, protein degradation, macrophages, leptin receptor antagonist

## Abstract

Obesity, a condition most commonly associated with hyper-leptinemia, is also characterized by increased expression of autophagy genes and likely autophagic activity in human adipose tissue (AT). Indeed, circulating leptin levels were previously shown to positively associate with the expression levels of autophagy genes such as Autophagy related gene-5 (ATG5). Here we hypothesized that leptin acts in an autocrine-paracrine manner to increase autophagy in two major AT cell populations, adipocytes and macrophages. We followed the dynamics of autophagosomes following acute leptin administration with or without a leptin receptor antagonist (SMLA) using high-throughput live-cell imaging in murine epididymal adipocyte and macrophage (RAW264.7) cell-lines. In macrophages leptin exerted only a mild effect on autophagy dynamics, tending to attenuate autophagosomes growth rate. In contrast, leptin-treated adipocytes exhibited a moderate, ~20% increase in the rate of autophagosome growth, an effect that was blocked by SMLA. This finding corresponded to mild increases in mRNA and protein expression of key autophagy genes. Interestingly, a long-lived proteins degradation assay uncovered a robust, >2-fold leptin-mediated stimulation of the autophagy/lysosome-related (bafilomycin-inhibited) activity, which was entirely blocked by SMLA. Collectively, leptin regulates autophagy in a cell-type specific manner. In adipocytes, autophagosome dynamics is moderately enhanced, but even more pronounced stimulation is seen in autophagy-related long-lived protein degradation. These findings suggest a causal link between obesity-associated hyperleptinemia and elevated adipocyte and AT autophagy-related processes.

## Introduction

Dys-regulation of autophagy, the evolutionarily-conserved processes by which cells degrade intracellular components to maintain cell homeostasis, is increasingly considered in the pathophysiology of various diseases.^1^ Although mostly dysfunctional (attenuated) autophagy is implicated in pathogenesis as a result of accumulated damaged or un-necessary cell components, over-activated autophagy is likely also pathogenic, and was proposed to contribute to the pathophysiology of several diseases, including chronic pulmonary disease and cancer.^2,3^ In obesity, autophagy is dys-regulated in a cell-type/tissue – specific manner. It is considered to be attenuated in the liver, altered in beta-cells, and decreased in skeletal and cardiac muscles.^4,5^ In adipose tissue, how autophagy is dys-regulated remains unsettled, with a general agreement that autophagy genes are over-expressed in adipose tissue, particularly in depots and in obesity sub-types that are more associated with metabolic dysfunction.^6–8^ Yet, although autophagy, particularly in chronic diseases, may be regulated also at the gene expression level,^9,10^ much more is known about its more rapid regulation by post-translational modifications and protein-protein interactions. Indeed, while two independent groups suggested that autophagic activity (i.e., ‘flux’) is inhibited in adipose tissue,^11,12^ at least four groups, including our own, provided evidence that the elevated gene expression of autophagy genes in adipose tissue manifests by increased autophagic flux.^6,13–16^ The molecular consequence of such elevated activity is not fully-mapped, but hyper-activated autophagy in adipose tissue may not only associate with,^17^ but can also be causally linked to, decreased secretion of adiponectin.^17,18^ This finding suggests a putative mechanistic link between activated adipose tissue autophagy in obesity, adipose tissue dysfunction, and a dys-metabolic obese phenotype.

Some outstanding questions emerge from the above: (1) since adipose tissue is composed of various cell types, what is the cell-specificity of autophagy alterations in obesity? Indeed, adipose tissue macrophages (ATM) may actually exhibit inhibited autophagy in obesity.^19^ Moreover, a transcriptional activator of adipose tissue autophagy in obesity, E2F1, is up-regulated in the adipocyte cell fraction but not in the stromal-vascular fraction of adipose tissue in obesity.^17^ (2) Among the various alterations that occur in the adipose tissue milieu in obesity, which is/are the factor(s) that dysregulate autophagy? Indeed, classically, autophagy is activated under conditions of nutrient deficiency. Inflammatory cytokines are common activators of autophagy in disease states, and although adipose tissue inflammation signifies metabolically-dysfunctional obesity, many of the proinflammatory cytokines are only mildly elevated in obesity compared to overt-inflammatory reaction. Circulating levels of leptin are robustly and positively associated with BMI,^20,21^ and the effect of leptin on autophagy was studied extensively in various cell types, and may be highly cell-type-specific: Leptin activated autophagy in muscle, heart, liver, kidney,^22^ conventional T cells^23^ and cancer cells,^24,25^ while inhibited autophagy in human lung cell line^26^ and recently was shown to inhibit ER-stress – induced autophagy in whole adipose tissue.^27^ A gap of knowledge remains in understanding whether and how leptin, produced by adipocytes at increased amounts in obesity, can regulate autophagy in adipocytes and other adipose tissue cell types.

In a previous study,^18^ we found that circulating leptin levels associate with visceral AT (VAT) expression of *ATG5* mRNA, a putative surrogate of adipose tissue autophagic activity. This led us to hypothesize that leptin, via the leptin receptor, may dys-regulate autophagy in a cell-type specific manner.

## Materials and methods

### Materials

Tissue culture medium (DMEM 01–055-1A, Biological Industries), heat-inactivated fetal bovine serum (04–121-1A, Biological Industries), antibiotic solutions (03–033-1B, Biological Industries), L-glutamine solutions (03–020-1B, Biological Industries), human recombinant insulin (01–818-1H, Biological Industries), phosphate-buffered saline (02–023-1A, Biological Industries) and Earle’s balanced salts solution (EBSS)(02–010-1A, Biological Industries). Indomethacin (I7378), dexamethasone (D4902), 3-isobutylmethylxanthine (IBMX; I7018), rosiglitazone (R2408) were obtained from Sigma-Aldrich. CYTO-ID and chloroquine (ENZ-51031, Enzo Life Science). BODIPY-C_12_ and Hoechst 33342 (D3835, H1399, Thermo Fisher Scientific,Inc). 3MA (M9281, Sigma-Aldrich). SMLA (SNAL-1, Protein Laboratories Rehovot Ltd).

### Cell culture

RAW264.7 murine macrophage cell line (American Type Culture Collection, Manassas, VA, USA) were cultured in DMEM 4.5 g/l glucose containing 10% FBS, 50 U/ml penicillin, 50 μg/ml streptomycin and 4 mM glutamine, as previously described.^19^

Epididymal pre-adipocyte cells^28,29^ were cultured as described. Briefly, cells were grown in DMEM 4.5 g/l glucose, supplemented with 20% FBS, 50 U/ml penicillin, 50 μg/ml streptomycin and 4 mM glutamine. Twenty-four h after reaching confluence, preadipocytes were induced to differentiate in media containing 0.125 mM indomethacin, 2 g/ml dexamethasone, and 0.5 mM 3-isobutylmethylxanthine for 48 h. For RT-PCR and western blot analysis, cells were seeded in 6-wells plates and experiments were held after 6 d of differentiation.

### Live imagining

For macrophages imagining, cells were cultured until they reached 60–80% confluence. Cells were scraped and 3 × 10^4^ cells were seeded in 96-well uClear black plate (Greiner Bio One, Kremsmünster, Austria). For adipocytes, on the third day after differentiation induction, cells were detached with trypsin. 4 × 10^4^ cells were seeded into 96-well uClear black plate.

3 h before treatments, the media was replaced to DMEM 4.5 g/l glucose containing 0.05% FBS, 50 U/ml penicillin, 50 μg/ml streptomycin and 4 mM glutamine. Cells were treated with or w/o leptin (10 and 100 ng/ml, orders of magnitude larger than endogenous leptin production from adipocytes cell-line (~2 pg/ml/h)), chloroquine (10 μmol/l). 3MA (5 mmol/l) and SLAN (50 μg/ml) were added to the media 30 minutes prior to the treatments mentioned above. For cell imaging and dynamic autophagosomes tracking, Hoechst (final dilution 1:1000) and CYTO-ID (final dilution 1:1000) were added to the media. For lipid droplets staining, BODIPY-C_12_ (1 µM) was used. Identification and quantification of images was done semi-automatically by Columbus software, and the mean value derived from each experiment was used to calculate the final comparison between experimental conditions.

### RNA extraction and quantitative real-time PCR

Total RNA was extracted using the RNeasy lipid tissue minikit (74804, Qiagen). Final concentration of 2000 ng/ml was used for reverse transcription into cDNA using reverse transcriptase kit (4374966, LifeTechnologies). cDNA was diluted 1:7 in ultra-pure water (002327777500, BioLab), amplified by the Taqman system (4369016, Life Technologies) and measured by RT-PCR (7500, Applied Biosystems). Relative gene expression was obtained after normalization to endogenous control genes (RPLP0 and HPRT). The following probes were used: Hprt (Mm03024075_m1), RPLP0 (Hs99999902_m1), Atg5 (Mm01187303_m1), Atg12 (Mm00503201_m1), Map1lc3b (Mm00782868_sH), LepR (total isoforms – extracellular domain) (NM_001122899.1) and LepR (long isoform) (NM_146146.2) (ThermoFisher Scientific,Inc).

### Cell lysates and western blot analysis

Western blot analysis was done as previously described.^17^Quantification was performed using GelQuant.NET software and normalized to those of ACTB. The following antibodies were used: Atg5, Atg7, Beclin-1 (2630, 2631, 3738, Cell signalling) and ACTB (A5441, Sigma-Aldrich)

### Autophagy-mediated long lived proteins degradation assay

We adopted a well-described protein degradation assay^30,31^ for use in our differentiated epididymal pre-adipocyte cells. Five days after differentiation induction, cells were ‘pulsed’ for 24 h with 50 µCi/ml L-[^14^C(U)]-Valine (NEC291EU050UC, PerkinElmer), then washed thoroughly, and incubated for the following 24 h period in medium containing 10 mM nonradioactive valine (V0513, Sigma-Aldrich). After an additional washout of the media with nonradioactive valine, the cells were ‘chased’ during a 6 h period in the above media, while being treated with: EBSS+0.1% BSA (= positive control for autophagy induction), control media (con), media containing leptin (100 ng/ml) only, or media containing leptin + SMLA (1 μg/ml). All conditions were given in the presence or absence of Baf.A. (0.1 μM), as indicated. SMLA was added 30 minutes prior to and during leptin treatment. A sample of 200 µl was taken at 2 h intervals and stored at −80◦ (200 µl of media was added back to the well). After 6 h, 700 µl were collected into tubes, the remaining media in the wells were removed, and wells were washed with cold PBS containing 10 mM valine. In order to separate the free amino-acids from the proteins, 20%TCA (T/2950/60, Fisher Scientific) was mixed with 1%PBS, 1:1 ratio, and was added to the media. Radioactivity was measured in the supernatant fractions (which contain mainly the free amino acids) and in the pellet of the cells (which contains mainly cellular proteins). % of [^14^C]-valine release was calculated as$=\frac{free\;animo\;acids\;radioactivity\;in\;the\;supernatant\;\times \;100}{total\;radioactivity\;\left(supernatant\;+\;cells^{\prime }pellet\right)}$

### Statistical analysis

Data are reported as mean +/- standard error of the mean (SEM). For two-groups comparison, none-parametric Mann-Whitney test was used. Comparison of> 2 groups was analysed after homogeneity of variances was confirmed using Levene test by one way analysis of variance (ANOVA) and the post-hoc Tukey’s method. When no equal variance was present, Kruskal-Wallis and Dunns tests were used. All statistical analyses and graphs were performed using GraphPad Prism 5. p < 0.05 was considered statistically significant.

## Results

**Figure 1.**
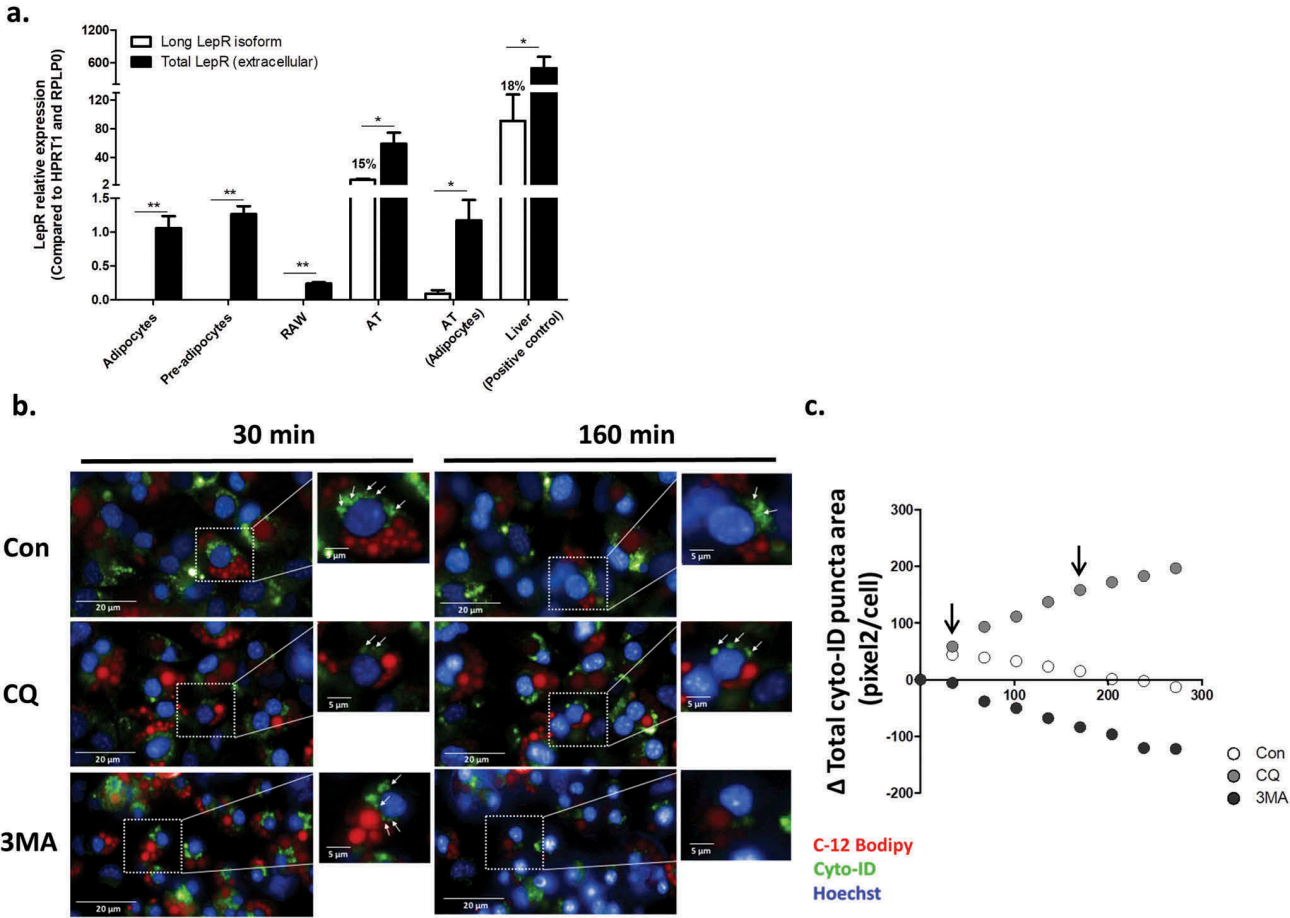
Leptin receptor isoforms expression and live-cell imaging of autophagosome dynamics in differentiated adipocytes. (a) The expression of total (black bars) and long (ObRb, white bars) LepR isoforms mRNA was measured in murine adipocyte cell-line (pre and after differentiation), RAW264.7 murine macrophages cell-line, mice adipose tissue (whole tissue and adipocytes fraction) and liver (positive control), using primers directed to the extracellular domain or the intracellular domain of ObRb, respectively. Results are presented as fold expression from adipocytes cell line (n = 4). Values are mean ± SEM. Means were compared by Mann-Whitney t-test. *p < 0.05, **p < 0.01. (b) Live-cell imaging (Operetta high throughput imaging system) of differentiated murine adipocyte cell-line stained with CYTO-ID (green) to detect autophagosomes (white arrows), in BODIPY-C_12_ (red, to stain lipid-droplets) – positive cells, and Hoechst (blue) to stain nuclei. Cells were treated with or without CQ (10 μmol/l) to inhibit autophagosome degradation, or with 3-methyladenine (3MA, 5 mmol/l) to inhibit autophagosome biogenesis. Scale bar, 20 μm and 5 μm in zoomed-in images. Shown are representative images. (c) Quantification of mean total autophagosome area per cell in BODIPY-C_12_ positive cells over time was done semi-automatically by following CYTO-ID punctae area using Columbus software. Black arrows correspond to the time-points shown in B.

Immune cell functions are altered by both changes in autophagy, and by leptin – an adipokine that signals through a cytokine receptor family member (the leptin receptor) that induces well-described immune-modulatory functions.^32^ Thus, we first assessed if obesity-related activation of adipose tissue autophagy could be attributed to the most abundant non-adipocyte cell type composing this tissue – i.e., macrophages.^33^ RAW 264.7 macrophages were treated with the lysosomal acidification inhibitor choloroquine to prevent degradation of newly-formed autophago(lyso)somes, and the dynamics of autophagosome formation and expansion (maturation) was studied using high-throughput live-cell imaging after staining cells with the fluorescent dye (CYTO-ID ©). CYTO-ID stains vesicles from the post-phagophore autophagosome stage to autophagolysosomes, irrespective of vesicular pH (but does not stain primary lysosomes), and is being used in a growing number of studies to detect autophagosome dynamics in live cells.^19,34,35^ Leptin exhibited only a mild effect on autophagy dynamics, tending to attenuate the initial rate of increase in total autophagosome area per cell (a composite measure of new autophagosome formation and expansion/maturation) (Figure S1(a,b)). This result was variable and not significant with 10 ng/ml (Figure S1(c)), but demonstrated a modest, though significant, ~15% decrease in the rate of total autophagosome area growth with 100 ng/ml. To determine the likelihood that this might represent a physiologically-relevant response, we measured LepR expression – both the total amount, and the long isoform (ObRb), using specific primers (detailed in Methods). Compared to adipocytes and to whole adipose tissue or liver samples, RAW264.7 cells expressed only minute amount of total LepR, but not its long isoform (Figure 1(a)). Thus, this cellular macrophage system provides only limited support for leptin’s effect on macrophage autophagy, and given the modest inhibitory effect – could not explain contribution to the activation of whole adipose tissue increased autophagy in obesity.

To estimate if leptin might activate autophagy in adipocytes, we first assessed LepR expression in the adipocyte cell line. Both pre-adipocytes and differentiated adipocytes expressed a readily-measurable amount of total LepR, but not the ObRb isoform. These levels were markedly lower than in whole adipose tissue (in which ObRb was also expressed, being 15% of the total LepR expression), but comparable to the expression of adipocyte fraction isolated from mouse adipose tissue after collagenase digestion (Figure 1(a)). Furthermore, leptin acutely increased AMPK phosphorylation in these cells (~30% increase at 5 min, an effect completely inhibited by the LepR inhibitor SMLA – data not shown). We next utilized the established live-cell imaging approach (used above in macrophages) to detect autophagosome dynamics in differentiated pre-adipocyte cell line (lipid droplets were stained by the fatty acid fluorescent probe BODIPY-C_12_). Over a > 4h tracking time, among the BODIPY-C_12_ positive cells (i.e., differentiated adipocytes), we observed that more autophagosomes accumulated in the cell when their degradation was prevented with choloroquine (Figure 1(b,c)). Conversely, the pan-PI3kinase inhibitor, 3MA, frequently used to inhibit autophagosome formation, markedly attenuated the process. Jointly, live-cell imaging of autophagy dynamics can be reliably achieved in differentiated adipocytes by tracking CYTO-ID-positive puncta.

**Figure 2.**
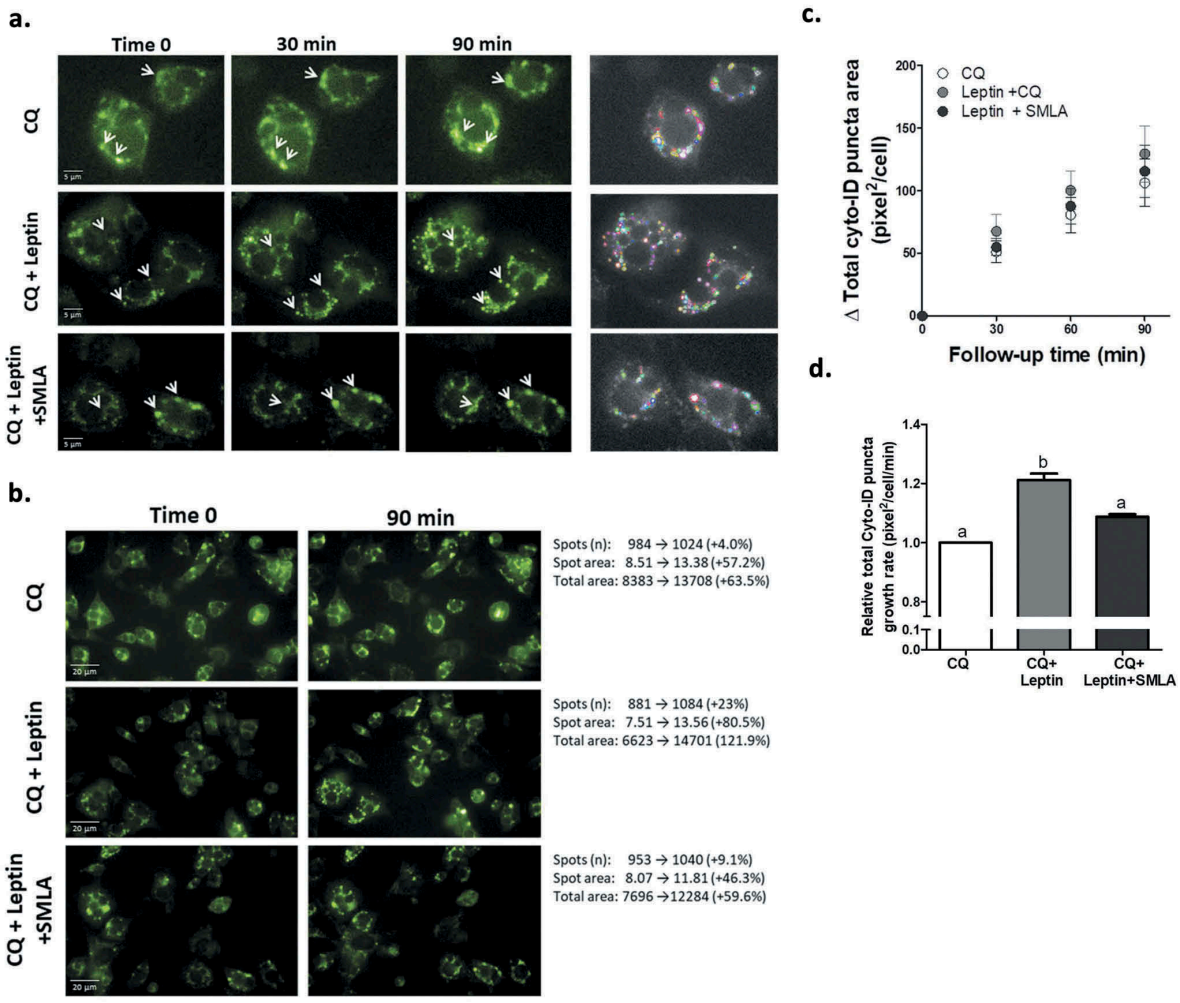
Leptin increases autophagosome area growth rate. Live cell imaging of differentiated murine adipocytes treated with leptin (100 ng/ml) without or with leptin receptor antagonist (SMLA 20 µg/ml – the latter was given 30 minute prior to recombinant leptin). CQ (10 μmol/l) was added as in Figure 1. Images are representative of three independent experiments performed in triplicates, with each well providing 23 images. Identification and quantification of images was done semi-automatically by Columbus software, and the mean value derived from each experiment was used to calculate the final comparison between experimental conditions. (a) Changes in CYTO-ID positive puncta (white arrows) in selected adipocytes over three time points. Right panel depicts the spots area as identified by the image analysis software. Scale bar, 5 μm. (b) Representative image (out of 23) and a summary of their changes in CYTO-ID spots parameters (spots number, spots area and total area) as measured by the software. Scale bar, 20 μm. (c) The change (∆ CYTO-ID positive puncta area) was calculated compared to the initial time-point from which CQ-mediated increase was observed (Follow-up time). (d) Growth rate (slope of graphs in C) of total cellular area of CYTO-ID positive puncta. ‘b’ = different from ‘a’ at confidence level of more than 95%.

**Figure 3.**
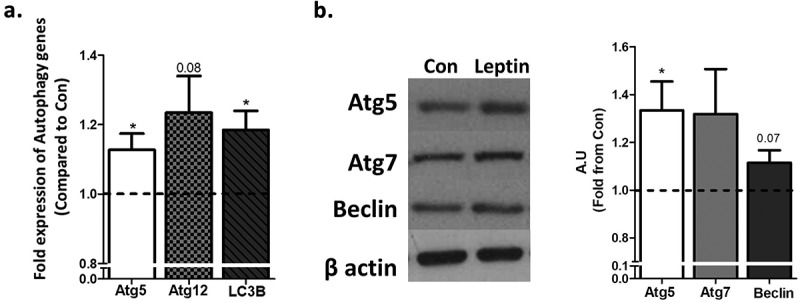
Leptin increases the expression of selected autophagy genes and proteins in adipocytes. Differentiated murine adipocytes were treated with leptin (100ng/ml) for 24h. (a) Autophagy related gene expression measured by RT-PCR, normalized to RPLP0 and HPRT. Results (n = 4 independent experiments) are the mean+SEM fold expression from control. (b) Representative Western blots of autophagy related proteins normalized to β actin. Results in the graph are the mean+SEM densitometry values of four independent experiments. *p < 0.05.

To assess leptin-induced changes in adipocyte autophagosomal “mass” (number and size), cells were pre-treated with chloroquine to prevent autophagosome degradation, and the change in autophagosome number and area was followed during the first 1.5 h of leptin treatment. As seen in individual cells quantified by the image analyses software (Figure 2(a)) and in whole representative fields (Figure 2(b)), Leptin induced a moderate, ~20% increase in the rate of autophagosome area/cell (Figure 2(c,d)), suggestive of increased autophagosome biogenesis and/or maturation/expansion. This effect was nearly fully inhibited by the leptin-R blocker, SMLA, confirming the specificity of the response to the leptin-LepR system. Since in adipose tissue in obesity autophagy genes’ mRNA and protein levels were increased, we also determined whether leptin could elevate the expression of such gene products in adipocytes. The mRNA levels of *ATG5* and of *Map1lc3b* were mildly but nevertheless significantly elevated (by ~15–20%), following stimulation of adipocytes with leptin (Figure 3(a)). A similar change was also detectable in some autophagy genes’ protein levels (Figure 3(b)).

**Figure 4.**
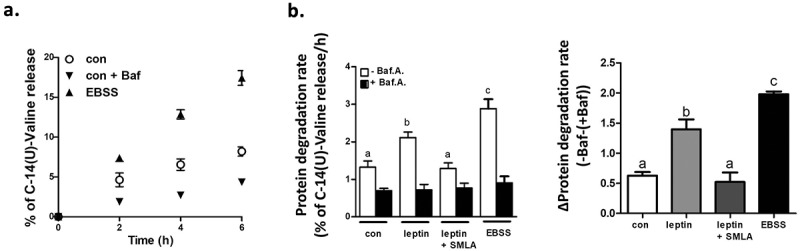
Leptin enhances long-lived protein degradation rate in adipocytes. (a) Time-dependent release of radioactive valine from epididymal adipocytes pre-pulsed with L-[^14^C(U)]-Valine, as described in Methods. Cells were either treated with EBSS+0.1% BSA to stimulate autophagy (= positive control), control media (con), media containing leptin (100 ng/ml) only, or media containing leptin + SMLA (1 μg/ml) with or without Baf.A. The radioactivity was measured in both the supernatant fraction and cell pellets and % of [^14^C]-valine release was calculated as described in Methods. (b) Long-lived protein degradation rate calculated as % of L-[^14^C(U)]-Valine release per 1 h, with or without Baf.A. (0.1 μM). Results are mean ± SEM of 3 independent experiments, each performed in triplicates. Different letters denote significant differences between treatments in the absence of Baf.A, p < 0.05. (c) Net autophagy-mediated L-[^14^C(U)]-Valine release (∆Protein degradation rate) as calculated from **b**. As in **b**, different letters denote significant differences between treatments (-Baf.A-(+Baf.A), p < 0.05.

Given the mild effect of leptin on adipocyte autophagy detected by the cell imaging system, we aimed to further assess whether leptin can activate autophagy-related functions, by adopting for use in adipocytes, an autophagy activity assay based on degradation of long-lived proteins.^31^ For this purpose we metabolically labelled long-lived proteins by pulsing cells with radiolabeled valine (a branched amino acid that does not affect mTOR, a regulator of autophagy), followed by a wash-out period of 24 h with media containing non-labelled valine, as detailed in methods. Over a 6 hour subsequent follow-up, ^14^C-valine release from long-lived proteins to the media was linear. More importantly, the autophagy inhibitor bafilomycin markedly inhibited, while serum and nutrient – free medium (EBSS) accelerated the rate of ^14^C-valine release (Figure 4(a)), consistent with a significant contribution of autophagy to this process. Non-autophagy long-lived protein degradation (i.e., in the presence of bafilomycin) was similar under all conditions (Figure 4(b), black bars). Nutrient- and serum-free buffer increased total ^14^C-valine release by >2-fold, and the rate of autophagy-mediated degradation by >3 fold (Figure 4(b,c)). Leptin treatment increased total and autophagy-mediated ^14^C-valine release (the latter by >2 fold), an effect that was completely blocked by the Leptin-R blocker SMLA (Figure 4(b,c)).

## Discussion

Although still debated, autophagy is likely activated in adipose tissue in obesity.^6,13–15,36,37^ The regulation of autophagy is highly cell and tissue – type, and biological context dependent, and indeed, multiple inducers of autophagy can be envisioned in the altered adipose tissue milieu in obesity. These include the development of adipose tissue hypoxia, inflammation, oxidative stress, ER stress, insulin resistance, etc.,^38^ although nutrient overload per-se (like high concentrations of free fatty acids and glucose) would theoretically tend to attenuate autophagy.^30,39^ In this study, we demonstrate that leptin can modestly modulate autophagosome dynamics as evident by the acceleration of the rate of growth of autophagosome area per cell – a composite measure of autophagosome number and size. This effect of leptin is evident in adipocytes, but not in the RAW macrophages. Although we demonstrate very low expression of LepR in these cells, others demonstrated leptin-induced activation of mTOR activity, which would inhibit autophagy.^40^ Utilizing autophagy-related degradation of long-lived proteins, leptin had a more pronounced activating effect in adipocytes. Taken together our results suggest that obesity-related hyperleptinemia may contribute to autophagy activation in adipose tissue, acting in an autocrine/paracrine manner via the leptin receptor.

Since autophagy is a dynamic process in which autophagosomes are generated and degraded, snap-shot analyses of cellular autophagosome number, size, and of autophagy-related proteins’ content can lead to misleading conclusions. An increase in autophagosomal ‘mass’ can result from increased biogenesis, and/or inhibited degradation of autophagosomes. Therefore, dynamic assays have become critical when evaluating autophagic activity, and ideally, this should be performed by more than a single assay.^41^ Here, we utilized dynamic, high throughput live-cell imaging to track the increase in autophagosome area when autophagolysosome degradation was inhibited. This allows assessing the effect of leptin on initial rate of autophagosome biogenesis and expansion (maturation). As stated above, leptin had a modest effect using this assay, as well as on the expression of several key autophagy gene mRNA and protein levels. Leptin’s capacity to activate autophagy was more robust when autophagic activity was assessed by the degradation rate of long-lived proteins. It is possible that the apparent differences of leptin’s efficacy to activate autophagy are due to different sensitivities of the two assays. Yet, it is also plausible that they uncover a true biological difference. Chaperone-mediated autophagy (CMA), and micro-autophagy, are two processes in which intracellular components, in particular proteins, are destined for degradation by the lysosomal compartment, by inserting them into the lysosome, independent of the creation of new autophagosomes that would need to merge with lysosomes to degrade their cargo.^42^ Indeed, degradation of the lipid droplet-associated proteins perilipin 2 and 3 was shown to be CMA-dependent.^43^ Thus, it is tempting to speculate that leptin may only modestly activate macro-autophagy, while more robustly augment CMA and/or micro-autophagy. Current literature provides little evidence, if any, on direct regulation of these processes by leptin: Chaperone-mediated autophagy was proposed to be activated by calorically restricting high-fat fed mice,^44^ a condition that would be predicted to diminish, not increase, leptin levels. Moreover, the effect was demonstrated in liver, a tissue in which macro-autophagy may be regulated opposite to adipose tissue in obesity.^38^ No information at all was found in the literature on leptin and micro-autophagy. Thus, it would seem worthwhile to address future studies on the potential capacity of leptin to particularly target one or both of these generally less-studied autophagic processes. This may be of particular relevance to adipocyte biology. In adipocytes 38% of detected proteome was reported to have an exceedingly long half-life, with no discernible degradation evident even after 96h of follow-up.^45^ Moreover, in that study, (macro)autophagy was proposed to contribute only minimally to total adipocyte protein degradation,^45^ suggesting that leptin, by particularly regulating other autophagy-type processes/lysosomal compartment – related process, could be an important regulator of adipocyte protein homeostasis.

It is noteworthy that a recent extensive study concluded that leptin inhibits ER-stress – induced autophagy.^27^ The *in-vivo* experiments of the study cannot exclude an indirect effect of leptin treatment, such as via its actions on hypothalamic centres. Yet, some in-vitro studies that also support the authors’ conclusions cannot be easily reconciled with our studies, though we propose that our highly dynamic approach to detect changes in autophagic flux, and more so – the finding that leptin may particularly augment long-lived protein degradation, possibly through mechanisms distinct from macroautophagy, may underlie the differences between the studies.

Obesity is thought to be associated with leptin resistance,^21^ questioning the relevance of our findings in obesity. Yet, whether adipocytes exhibit leptin resistance is not unclear: several papers concluded that obesity induces leptin resistance in whole AT,^46,47^ characterized by decreased pSTAT3 levels and elevated SOCS-3 expression. Yet, other studies^27^ do show leptin responsiveness of AT in obese (ob/ob) mice. Regardless, the chronicity of obesity may suggest that any leptin regulation of adipocyte autopahgic process as we show here, even if with a small effect size due to LepR expression and/or to obesity-related leptin resistance, may nevertheless bear physiological relevance over time.

Leptin is only one, out of hundreds of peptides, which are altered in obesity. Yet, since adipocytes are its major source, and given that obesity is usually associated with hyperleptinemia, local concentrations of leptin in adipose tissue may be very high. Systemically, leptin’s circulating levels are amongst the adipocytokines with the highest dynamic range, particularly differentiating lean from obese persons. Thus, despite being a single factor amongst many, which is studied here in isolation, it is not impossible that leptin contributes significantly to the dysregulated/activated adipose tissue autophagy and to this tissue’s dysfunction in obesity.

## References

[1] Choi AM, Ryter SW, Levine B. Autophagy in human health and disease. N Engl J Med. 2013 2 14;368(7):651–662. doi:10.1056/NEJMra1205406 PubMed PMID: 23406030. 23406030

[2] Chen Z-H, Kim HP, Sciurba FC, Lee S-J, Feghali-Bostwick C, Stolz DB, Dhir R, Landreneau RJ, Schuchert MJ, Yousem SA, et al Egr-1 regulates autophagy in cigarette smoke-induced chronic obstructive pulmonary disease. PLoS One 2008;3(10):e3316. doi:10.1371/journal.pone.0003316 PubMed PMID: 18830406; PubMed Central PMCID: PMC2552992. 18830406 PMC2552992

[3] Yang S, Wang X, Contino G, Liesa M, Sahin E, Ying H, Bause A, Li Y, Stommel JM, Dell’antonio G, et al Pancreatic cancers require autophagy for tumor growth. Genes Dev. 2011 4 1;25(7):717–729. doi:10.1101/gad.2016111 PubMed PMID: 21406549; PubMed Central PMCID: PMCPMC3070934. 21406549 PMC3070934

[4] Stienstra R, Haim Y, Riahi Y, Netea M, Rudich A, Leibowitz G. Autophagy in adipose tissue and the beta cell: implications for obesity and diabetes. Diabetologia. 2014 8;57(8):1505–1516. doi:10.1007/s00125-014-3255-3 PubMed PMID: 24795087. 24795087

[5] van Niekerk G, du Toit A, Loos B, Engelbrecht A-M Nutrient excess and autophagic deficiency: explaining metabolic diseases in obesity. Metabolism. 2018 5;82:14–21. doi:10.1016/j.metabol.2017.12.007 PubMed PMID: 29289514. 29289514

[6] Kovsan J, Blüher M, Tarnovscki T, Klöting N, Kirshtein B, Madar L, Shai I, Golan R, Harman-Boehm I, Schön MR, et al Altered autophagy in human adipose tissues in obesity. J Clin Endocrinol Metab. 2011 2;96(2):E268–E77. doi:10.1210/jc.2010-1681 PubMed PMID: 21047928. 21047928

[7] Xu Q, Mariman ECM, Roumans NJT, Vink RG, Goossens GH, Blaak EE, Jocken JWE Adipose tissue autophagy related gene expression is associated with glucometabolic status in human obesity. Adipocyte. 2018 1 2;7(1):12–19. doi:10.1080/21623945.2017.1394537 PubMed PMID: 29400609; PubMed Central PMCID: PMCPMC5915036. 29400609 PMC5915036

[8] Zhang Y, Sowers JR, Ren J Targeting autophagy in obesity: from pathophysiology to management. Nat Rev Endocrinol. 2018 6;14(6):356–376. doi:10.1038/s41574-018-0009-1 10.1038/s41574-018-0009-1. PubMed PMID: 29686432. 29686432

[9] Maixner N, Bechor S, Vershinin Z, Pecht T, Goldstein N, Haim Y, Rudich A Transcriptional dysregulation of adipose tissue autophagy in obesity. Physiology. 2016 7;31(4):270–282. doi:10.1152/physiol.00048.2015 PubMed PMID: 27252162. 27252162

[10] Fullgrabe J, Klionsky DJ, Joseph B The return of the nucleus: transcriptional and epigenetic control of autophagy. Nat Rev Mol Cell Biol. 2014 1;15(1):65–74. doi:10.1038/nrm3716 PubMed PMID: 24326622. 24326622

[11] Soussi H, Reggio S, Alili R, Prado C, Mutel S, Pini M, Rouault C, Clément K, Dugail I DAPK2 downregulation associates with attenuated adipocyte autophagic clearance in human obesity. Diabetes. 2015 10;64(10):3452–3463. doi:10.2337/db14-1933 PubMed PMID: 26038578. 26038578

[12] Yoshizaki T, Kusunoki C, Kondo M, Yasuda M, Kume S, Morino K, Sekine O, Ugi S, Uzu T, Nishio Y, et al Autophagy regulates inflammation in adipocytes. Biochem Biophys Res Commun. 2012;417:352–357. doi:10.1016/j.bbrc.2011.11.114. 22155234

[13] Ost A, Svensson K, Ruishalme I, Brännmark C, Franck N, Krook H, Sandström P, Kjolhede P, Strålfors P Attenuated mTOR signaling and enhanced autophagy in adipocytes from obese patients with type 2 diabetes. Mol Med. 2010 Jul-Aug;16(7–8):235–246. doi:10.2119/molmed.2010.00023 PubMed PMID: 20386866; PubMed Central PMCID: PMCPMC2896460. 20386866 PMC2896460

[14] Jansen HJ, van Essen P, Koenen T, Joosten LAB, Netea MG, Tack CJ, Stienstra R Autophagy activity is up-regulated in adipose tissue of obese individuals and modulates proinflammatory cytokine expression. Endocrinology. 2012 12;153(12):5866–5874. doi:10.1210/en.2012-1625 PubMed PMID: 23117929. 23117929

[15] Nuñez CE, Rodrigues VS, Gomes FS, de Moura RF, Victorio SC, Bombassaro B, Chaim EA, Pareja JC, Geloneze B, Velloso LA, et al Defective regulation of adipose tissue autophagy in obesity. Int J Obes. 2013 11;37(11):1473–1480. doi:10.1038/ijo.2013.27 PubMed PMID: 23478428. 23478428

[16] Kosacka J, Nowicki M, Paeschke S, Baum P, Blüher M, Klöting N Up-regulated autophagy: as a protective factor in adipose tissue of WOKW rats with metabolic syndrome. Diabetol Metab Syndr. 2018;10:13. doi:10.1186/s13098-018-0317-6 PubMed PMID: 29507613; PubMed Central PMCID: PMCPMC5834836. 29507613 PMC5834836

[17] Haim Y, Blüher M, Slutsky N, Goldstein N, Klöting N, Harman-Boehm I, Kirshtein B, Ginsberg D, Gericke M, Guiu Jurado E, et al Elevated autophagy gene expression in adipose tissue of obese humans: A potential non-cell-cycle-dependent function of E2F1. Autophagy. 2015 9 22;11:2074–2088. doi:10.1080/15548627.2015.1094597 PubMed PMID: 26391754. 26391754 PMC4824599

[18] Slutsky N, Vatarescu M, Haim Y, Goldstein N, Kirshtein B, Harman-Boehm I, Gepner Y, Shai I, Bashan N, Blüher M, et al Decreased adiponectin links elevated adipose tissue autophagy with adipocyte endocrine dysfunction in obesity. Int J Obes. 2016 6;40(6):912–920. doi:10.1038/ijo.2016.5 PubMed PMID: 26786352. 26786352

[19] Bechor S, Nachmias D, Elia N, Haim Y, Vatarescu M, Leikin-Frenkel A, Gericke M, Tarnovscki T, Las G, Rudich A Adipose tissue conditioned media support macrophage lipid-droplet biogenesis by interfering with autophagic flux. Biochim Biophys Acta. 2017 9;1862(9):1001–1012. doi:10.1016/j.bbalip.2017.06.012 PubMed PMID: 28652194. 28652194

[20] Maffei M, Halaas J, Ravussin E, Pratley RE, Lee GH, Zhang Y, Fei H, Kim S, Lallone R, Ranganathan S Leptin levels in human and rodent: measurement of plasma leptin and ob RNA in obese and weight-reduced subjects. Nat Med. 1995 11;1(11):1155–1161. PubMed PMID: 7584987. 7584987 10.1038/nm1195-1155

[21] Considine RV, Sinha MK, Heiman ML, Kriauciunas A, Stephens TW, Nyce MR, Ohannesian JP, Marco CC, McKee LJ, Bauer TL Serum immunoreactive-leptin concentrations in normal-weight and obese humans. N Engl J Med. 1996 2 1;334(5):292–295. doi:10.1056/NEJM199602013340503 PubMed PMID: 8532024. 8532024

[22] Malik SA, Mariño G, BenYounès A, Shen S, Harper F, Maiuri MC, Kroemer G Neuroendocrine regulation of autophagy by leptin. Cell Cycle. 2011 9 1;10(17):2917–2923. doi:10.4161/cc.10.17.17067 PubMed PMID: 21857156. 21857156

[23] Cassano S, Pucino V, La Rocca C, Procaccini C, De Rosa V, Marone G, Matarese G Leptin modulates autophagy in human CD4+CD25- conventional T cells. Metabolism. 2014 10;63(10):1272–1279. doi:10.1016/j.metabol.2014.06.010 PubMed PMID: 25060689; PubMed Central PMCID: PMCPMC4180014. 25060689 PMC4180014

[24] Raut PK, Choi DY, Kim SH, Hong JT, Kwon TK, Jeong JH, Park P-H Estrogen receptor signaling mediates leptin-induced growth of breast cancer cells via autophagy induction. Oncotarget. 2017 12 12;8(65):109417–109435. doi:10.18632/oncotarget.22684 PubMed PMID: WOS:000419565400090; English. 29312618 PMC5752531

[25] Nepal S, Kim MJ, Hong JT, Kim SH, Sohn D-H, Lee SH, Song K, Choi DY, Lee ES, Park P-H Autophagy induction by leptin contributes to suppression of apoptosis in cancer cells and xenograft model: involvement of p53/FoxO3A axis. Oncotarget. 2015 3 30;6(9):7166–7181. doi:10.18632/oncotarget.3347 PubMed PMID: 25704884; PubMed Central PMCID: PMCPMC4466676. 25704884 PMC4466676

[26] Gui X, Chen H, Cai H, Sun L, Gu L Leptin promotes pulmonary fibrosis development by inhibiting autophagy via PI3K/Akt/mTOR pathway. Biochem Biophys Res Commun. 2018 4 6;498(3):660–666. doi:10.1016/j.bbrc.2018.03.039 PubMed PMID: 29524411. 29524411

[27] Gan L, Liu Z, Luo D, Ren Q, Wu H, Li C, Sun C Reduced endoplasmic reticulum stress-mediated autophagy is required for leptin alleviating inflammation in adipose tissue. Front Immunol. 2017;8:1507. doi:10.3389/fimmu.2017.01507 PubMed PMID: 29250056; PubMed Central PMCID: PMCPMC5715390. 29250056 PMC5715390

[28] Klein J, Fasshauer M, Ito M, Lowell BB, Benito M, Kahn CR beta(3)-adrenergic stimulation differentially inhibits insulin signaling and decreases insulin-induced glucose uptake in brown adipocytes. J Biol Chem. 1999 12 3;274(49):34795–34802. PubMed PMID: 10574950. 10574950 10.1074/jbc.274.49.34795

[29] Kovsan J, Osnis A, Maissel A, Mazor L, Tarnovscki T, Hollander L, Ovadia S, Meier B, Klein J, Bashan N, et al Depot-specific adipocyte cell lines reveal differential drug-induced responses of white adipocytes–relevance for partial lipodystrophy. Am J Physiol Endocrinol Metab. 2009 2;296(2):E315–E22. doi:10.1152/ajpendo.90486.2008 PubMed PMID: 19033543. 19033543

[30] Las G, Serada SB, Wikstrom JD, Twig G, Shirihai OS Fatty acids suppress autophagic turnover in beta-cells. J Biol Chem. 2011 12 9;286(49):42534–42544. doi:10.1074/jbc.M111.242412 PubMed PMID: 21859708; PubMed Central PMCID: PMCPMC3234912. 21859708 PMC3234912

[31] Bauvy C, Meijer AJ, Codogno P Assaying of autophagic protein degradation. Methods Enzymol. 2009;452:47–61. doi:10.1016/S0076-6879(08)03604-5 PubMed PMID: 19200875. 19200875

[32] Pérez-Pérez A, Vilariño-García T, Fernández-Riejos P, Martín-González J, Segura-Egea JJ, Sánchez-Margalet V Role of leptin as a link between metabolism and the immune system. Cytokine Growth Factor Rev. 2017 6;35:71–84. doi:10.1016/j.cytogfr.2017.03.001 PubMed PMID: 28285098. 28285098

[33] Weisberg SP, McCann D, Desai M, Rosenbaum M, Leibel RL, Ferrante AW Obesity is associated with macrophage accumulation in adipose tissue. J Clin Invest. 2003 12;112(12):1796–1808. doi:10.1172/JCI19246 PubMed PMID: 14679176; PubMed Central PMCID: PMCPMC296995. 14679176 PMC296995

[34] Lima S, Takabe K, Newton J, Saurabh K, Young MM, Leopoldino AM, Hait NC, Roberts JL, Wang H-G, Dent P, et al TP53 is required for BECN1- and ATG5-dependent cell death induced by sphingosine kinase 1 inhibition. Autophagy. 2018 3 11;1–16. doi:10.1080/15548627.2018.1429875 PubMed PMID: 29368980. PMC6103396 29368980

[35] Chan LL-Y, Shen D, Wilkinson AR, Patton W, Lai N, Chan E, Kuksin D, Lin B, Qiu J A novel image-based cytometry method for autophagy detection in living cells. Autophagy. 2012 9;8(9):1371–1382. doi:10.4161/auto.21028 PubMed PMID: 22895056; PubMed Central PMCID: PMCPMC3442883. 22895056 PMC3442883

[36] Kosacka J, Kern M, Klöting N, Paeschke S, Rudich A, Haim Y, Gericke M, Serke H, Stumvoll M, Bechmann I, et al Autophagy in adipose tissue of patients with obesity and type 2 diabetes. Mol Cell Endocrinol. 2015;409:21–32. doi:10.1016/j.mce.2015.03.015. 25818883

[37] Li H, Zhou B, Xu L, Liu J, Zang W, Wu S, Sun H The reciprocal interaction between autophagic dysfunction and ER stress in adipose insulin resistance. Cell Cycle 2014;13(4):565–579. doi:10.4161/cc.27406 PubMed PMID: 24309597. 24309597

[38] Maixner N, Kovsan J, Harman-Boehm I, Blüher M, Bashan N, Rudich A Autophagy in adipose tissue. Obes Facts 2012;5(5):710–721. doi:10.1159/000343983 PubMed PMID: 23108431. 23108431

[39] Mir SU, George NM, Zahoor L, Harms R, Guinn Z, Sarvetnick NE Inhibition of autophagic turnover in beta-cells by fatty acids and glucose leads to apoptotic cell death. J Biol Chem. 2015 3 6;290(10):6071–6085. doi:10.1074/jbc.M114.605345 PubMed PMID: 25548282; PubMed Central PMCID: PMCPMC4358249. 25548282 PMC4358249

[40] Maya-Monteiro CM, Almeida PE, D’Avila H, Martins AS, Rezende AP, Castro-Faria-Neto H, Bozza PT. Leptin induces macrophage lipid body formation by a phosphatidylinositol 3-kinase- and mammalian target of rapamycin-dependent mechanism. J Biol Chem. 2008 Jan 25;283(4):2203–2210. doi: 10.1074/jbc.M706706200. PubMed PMID: 18039669. 18039669

[41] Klionsky DJ, Abdelmohsen K, Abe A, et al Guidelines for the use and interpretation of assays for monitoring autophagy. Autophagy. 2016;12(1):1–222. 3rd edition. doi:10.1080/15548627.2015.1100356 PubMed PMID: 26799652; PubMed Central PMCID: PMCPMC4835977. 26799652 PMC4835977

[42] Cuervo AM, Wong E Chaperone-mediated autophagy: roles in disease and aging. Cell Res. 2014 1;24(1):92–104. doi:10.1038/cr.2013.153 PubMed PMID: 24281265; PubMed Central PMCID: PMCPMC3879702. 24281265 PMC3879702

[43] Kaushik S, Cuervo AM Degradation of lipid droplet-associated proteins by chaperone-mediated autophagy facilitates lipolysis. Nat Cell Biol. 2015 6;17(6):759–770. doi:10.1038/ncb3166 PubMed PMID: 25961502; PubMed Central PMCID: PMCPMC4449813. 25961502 PMC4449813

[44] Schloesser A, Campbell G, Glüer C-C, Rimbach G, Huebbe P Restriction on an energy-dense diet improves markers of metabolic health and cellular aging in mice through decreasing hepatic mTOR activity. Rejuvenation Res. 2015 2;18(1):30–39. doi:10.1089/rej.2014.1630 PubMed PMID: 25405871; PubMed Central PMCID: PMCPMC4340804. 25405871 PMC4340804

[45] Minard AY, Wong MKL, Chaudhuri R, Tan S-X, Humphrey SJ, Parker BL, Yang JY, Laybutt DR, Cooney GJ, Coster ACF, et al Hyperactivation of the insulin signaling pathway improves intracellular proteostasis by coordinately up-regulating the proteostatic machinery in adipocytes. J Biol Chem. 2016 12 2;291(49):25629–25640. doi:10.1074/jbc.M116.741140 PubMed PMID: 27738101; PubMed Central PMCID: PMCPMC5207260. 27738101 PMC5207260

[46] Wang Z, Zhou YT, Kakuma T, Lee Y, Kalra SP, Kalra PS, Pan W, Unger RH Leptin resistance of adipocytes in obesity: role of suppressors of cytokine signaling. Biochem Biophys Res Commun. 2000 10 14;277(1):20–26. doi:10.1006/bbrc.2000.3615 PubMed PMID: 11027633. 11027633

[47] Wang M-Y, Orci L, Ravazzola M, Unger RH Fat storage in adipocytes requires inactivation of leptin’s paracrine activity: implications for treatment of human obesity. Proc Natl Acad Sci U S A. 2005 12 13;102(50):18011–18016. doi:10.1073/pnas.0509001102 PubMed PMID: 16326804; PubMed Central PMCID: PMCPMC1312408. 16326804 PMC1312408

