# Supplementary figures and images for: Leptin stimulates autophagy/lysosome-related degradation of long-lived proteins in adipocytes
Source: Adipocyte. 2019 Feb 8;8(1):51–60. doi: 10.1080/21623945.2019.1569447 (PMC6768270; doi:10.1080/21623945.2019.1569447)

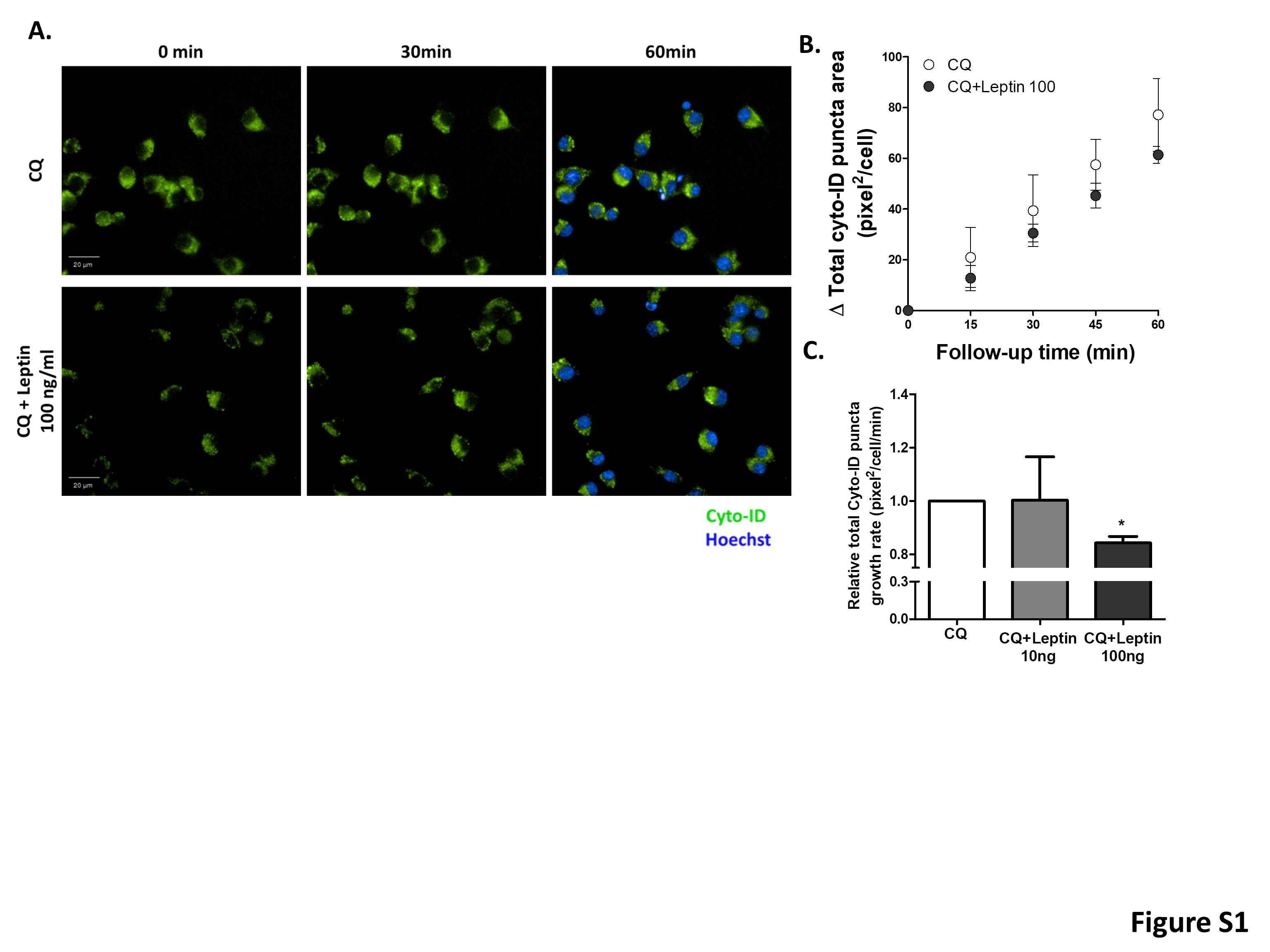

Supplement: Supplemental Material [file kadi-08-01-1569447-s001.jpg]
